# Multifocal extranodal lymphoma: A case report

**DOI:** 10.1097/MD.0000000000005029

**Published:** 2016-10-07

**Authors:** Chao Li, Lin Li, Ping Zhang, Jin-Song Zhang, Ting Gao, Yan Xu, Wen-Chan Li

**Affiliations:** a Department of Oncology, Beijing Hospital; b Department of Pathology, Beijing Hospital; c Cancer Hospital, Chinese Academy of Medical Science; d Department of Nuclear Medicine, Beijing Hospital, Beijing, China.

**Keywords:** central nervous system involvement, diffuse large B cell lymphoma, multifocal extranodal lymphoma, positron emission tomography/computed tomography

## Abstract

**Introduction:**

We report an unusual and interesting case of non-Hodgkin lymphoma involving 7 extranodal sites.

In this case, a 43-year-old woman with diffuse large B-cell lymphoma, including stomach, breasts, pancreas, adrenal glands, ovary and bones, was confirmed by biopsy and positron emission tomography/computed tomography scan. The patient achieved a complete response after 2 cycles of chemotherapy with combined rituximab, cyclophosphamide, doxorubicin, vincristine, and prednisolone, but subsequently developed central nervous system involvement.

**Conclusion:**

This case illustrated the usefulness of positron emission tomography/computed tomography in diagnosis, disease staging, and assessment of response to therapy. Selection of the optimal treatment regimen is challenging and needs further research.

## 1 Introduction

Non-Hodgkin lymphoma (NHL) arises primarily in sites other than lymph nodes, including extranodal tissues.^[1,2]^ Extranodal lymphoma can arise in any organ, and can multifocally affect 2 or more organs. Here, we present an uncommon and rarely reported disease pattern: a 43-year-old woman with confirmed multifocal extranodal lymphoma of the stomach, breasts, pancreas, adrenal glands, ovary and bones, followed by central nervous system (CNS) involvement. The study protocol was reviewed and approved by Ethics Committee of Beijing Hospital.

Written informed consent was obtained from the husband of the patient.

## 2 Case report

A 43-year-old female who had been well until 2 months before presenting as a patient developed mid-epigastric pain, radiating to the back, weight loss, nausea, and vomiting. She was admitted to our hospital on March 13, 2015. She had no history of fever or night sweats, or any contributory medical or family history. Her physical examination on admission showed blood pressure: 112/78 mm Hg; pulse rate: 70/min; respiration rate: 18/min; and body temperature: 36.5°C. Tonsils were not swollen, and palpation showed no enlargement of cervical, axillary, or inguinal lymph nodes. Intraoral inspection found a solitary swelling involving the mandible mucosa. Both breasts seemed swollen, but no specific mass was observed. She had direct tenderness on her upper abdomen, without rebound tenderness, but no hepatomegaly or splenomegaly.

Apart from elevated lactate dehydrogenase (LDH) level (1553 U/L [normal: 109–245 U/L]), β2-microglobulin (4.86 mg/L [normal: 0.7–1.8 mg/L]), amylase (364 U/L [normal: 28–100 U/L]), and low hemoglobin (85 g/L [normal: 110–160 g/L]), laboratory tests, including hepatitis B and C virus and human immunodeficiency virus, were unremarkable.

**Figure 1:**
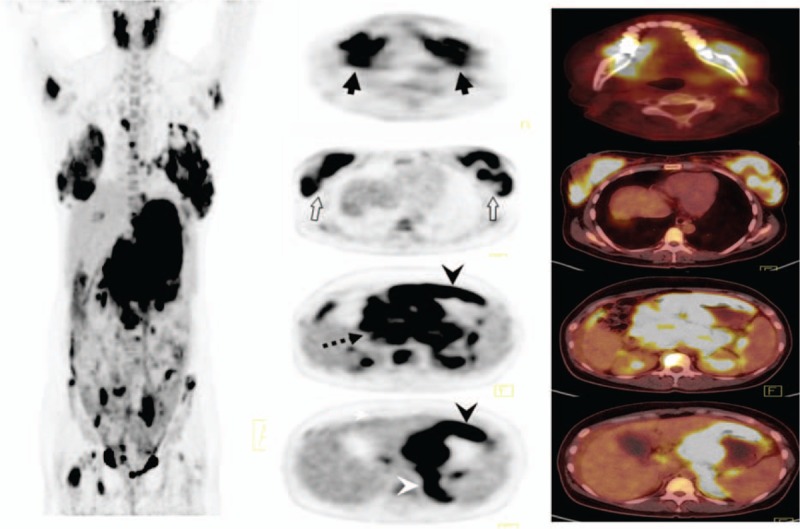
PET/CT images. PET/CT scan showed multiple hypermetabolic lesions in both mandible (black arrows), both breasts (white arrows), stomach (black arrowhead), pancreas (dotted arrow), and left adrenal (white arrowhead). PET/CT = positron emission tomography/computed tomography.

**Figure 2:**
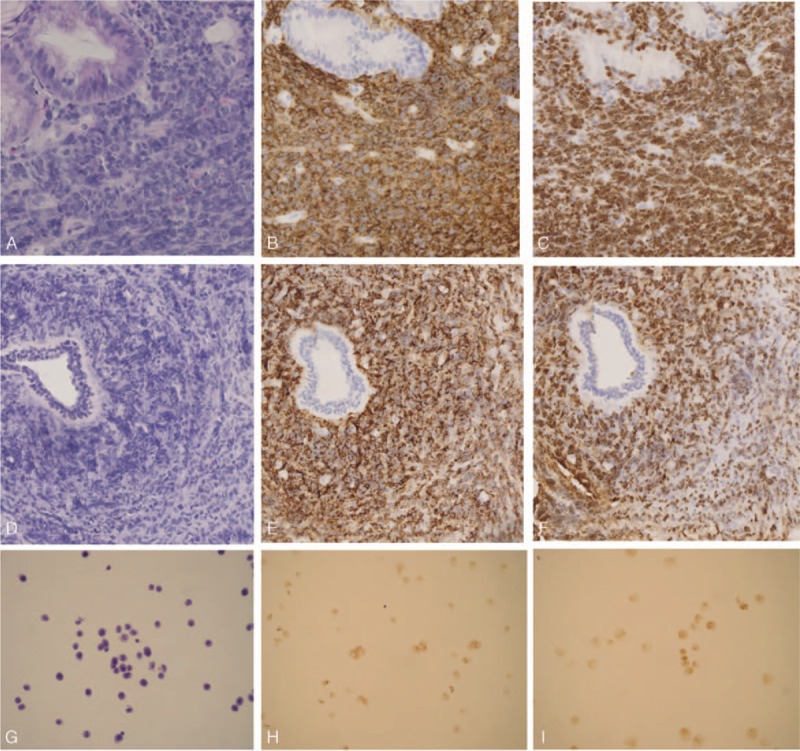
H&E staining and immunohistochemical characteristics of tumor cells. A, D, Hematoxylin and eosin (H&E) staining revealed diffuse infiltration with large atypical lymphoid cells with prominent nucleoli. A, H&E staining of the endoscopic biopsy (×200). B, CD20-positive malignant cells from endoscopic biopsy (×100). C, Ki67 strongly positive malignant cells from endoscopic biopsy (×100). D, H&E staining of the breast biopsy (×100). E, CD20-positive malignant cells from breast biopsy (×100). F, Ki67 strongly positive malignant cells from breast biopsy (×100). G, H&E staining of cerebrospinal fluid (CSF) smear revealed atypical lymphoid cells (×100). H, CD20-positive malignant cells from CSF (×100). I, Ki67-positive malignant cells from CFS (×100). CSF = cerebrospinal fluid.

Chest computed tomography (CT) scans showed no visible masses. Abdominal CT scan showed abnormally thickened gastric walls with perigastric lymph adenopathy and enlargement of the pancreatic head. Positron emission tomography/computed tomography (PET/CT) scan showed perigastric lymph adenopathy and multiple hypermetabolic lesions in stomach, pancreas, both breasts, left adrenal gland, left ovary, and multiple bones including mandibles and femur (Fig. 1). Histopathological examinations of individually performed endoscopic and breast biopsies led to the diagnosis of diffuse large B-cell lymphoma (DLBCL). Immunohistological examination of tumor cells indicated them as endoscopy: CD20^+++^, CD3^+^, CD10^−^, Bcl6^-^, Mum1^++^, Ki67 (80%), CD21^−^, AE1/AE3^+^, CgA^−^; and breast: CD20^+++^, CD3^+^, CD10^−^, Bcl6^+^, Mum1^+^, Ki67 (80%) (Fig. 2A–F). These findings were consistent with imaging that showed multifocal extranodal DLBCL involving the stomach, breasts, pancreas, adrenal glands, ovary, and bones. The patient was diagnosed with stage IVB DLBCL and an international prognostic index (IPI) score of 4 (high risk).^[3]^

After 2 chemotherapy cycles (21 days each) of combined rituximab, cyclophosphamide, doxorubicin, vincristine, and prednisolone (R-CHOP), a follow-up PET/CT scan showed complete metabolic resolution of lesions in her stomach, breasts, pancreas, adrenal, ovary, and bones, indicating excellent therapeutic efficacy. The epigastric pain and swelling of her jaw and breasts were evidently relieved. She underwent a third R-CHOP chemotherapy cycle.

**Figure 3:**
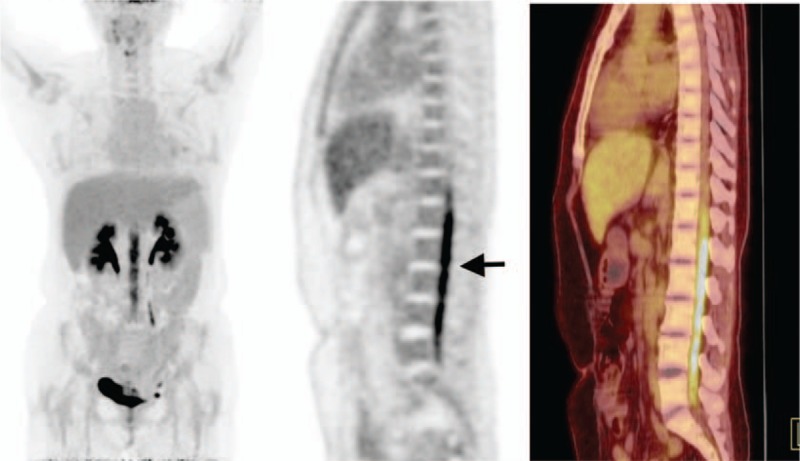
PET/CT images. After 3 cycles of R-CHOP chemotherapy, PET/CT scan showed hypermetabolic lesion in T12-L5 vertebral levels of the spinal cord (*arrow*).

However, about 4 months after her diagnosis, the patient developed headache, nausea, and vomiting. Although brain MRI and CT scans were normal, PET/CT showed a hypermetabolic lesion at her T12-L5 vertebral level (Fig. 3). Cerebrospinal fluid cytology indicated that her DLBCL had returned; immunohistochemistry showed CD79α^−^, Mum1^+^, CD20 ^++^ tumor cells with a highly proliferative Ki67 index (Fig. 2G–I). This patient's symptoms reflected rapidly increasing intracranial pressure. Sadly, she died after 2 weeks.

## 3 Discussion

DLBCL is the most prevalent NHL subtype. Extranodal NHL reportedly accounts for approximately one-third of NHL cases,^[4,5]^ and incidence of extranodal NHL is increasing worldwide, especially in Western countries.^[6,7]^ Although extranodal lymphomas are not rare, concurrent involvement of the stomach, breasts, pancreas, adrenal glands, ovary, and bones in a multifocal extranodal lymphoma is very uncommon.^[1,2]^

Clinical symptoms of lymphoma are nonspecific. This patient presented with epigastric pain, weight loss, nausea, vomiting, swollen jaw, and swollen breasts, which were collectively difficult to differentiate from other benign and malignant lesions.

Many of the CT scan and endoscopic findings (such as thickened gastric walls) were also nonspecific and might have been seen in other common tumors. However, this case showed the imaging features of a rare multifocal extranodal DLBCL, and highlighted the power of PET/CT scans in assessing the extensive disease involvement. PET/CT scans are also used in lymphoma to guide biopsies and evaluate treatment response.^[8]^ Ultimately, however, the confirmatory diagnosis was based on pathologic examination; endoscopic biopsy, breast biopsy, and cerebrospinal fluid cytology played a very important role in the diagnosis of DLBCL.

Treatment strategies for DLBCL patients differ for localized (Ann Arbor stages I–II) and advanced (Ann Arbor stages III–IV) disease, and also depend on a patient's age, physical condition, histological subtype, comorbidity, burden of disease, and other factors. The R-CHOP chemotherapy regimen has been the standard treatment for patients with advanced-stage DLBCL.^[9–12]^ In this case, the patient achieved a complete response after 2 cycles of R-CHOP chemotherapy.

Approximately 20% of patients with systemic lymphoma reportedly develop CNS involvement,^[13]^ and approximately 95% of CNS lymphoma cases are DLBCL.^[14]^ The increasing incidence of CNS lymphoma may be partly due to immunodeficiency following multiregimen chemotherapy, but can also be attributed to standard lymphoma regimens (such as CHOP) which fail to penetrate blood–brain barrier.^[15]^ Moreover, some patients with paranasal sinus involvement or who have 2 or more extranodal sites with elevated LDH are vulnerable to CNS involvement.^[16–19]^ Therefore, prophylactic intrathecal injections with high doses of methotrexate and/or cytarabine are recommended for those patients.^[20,21]^ CNS progression in NHL patients is associated with pessimistic outcomes.^[22]^ Reported median survival periods for NHL patients with CNS involvement are, for CNS involvement found at diagnosis: 5.4 months; found at disease recurrence: 3.8 months; and for CNS involvement that progresses during treatment: 1.8 months.^[23]^

Currently, the most frequently used and important clinical predictive system for patients with lymphoma is the IPI score, which takes age, Ann Arbor stage, LDH level, performance status, and the number of extranodal disease sites into account.^[24]^ In addition to her CNS involvement, this patient's IPI score was 4, which indicates poor prognosis.

In conclusion, we have presented a rare, multifocal extranodal DLBCL involvement of 7 anatomical sites. A PET/CT scan can initially find, and greatly characterize such an uncommon multifocal extranodal DLBCL presentation, and evaluate the extent of disease involvement, and later assess the efficacy of the treatment. However, biopsy or surgery is the key to the diagnosis of DLBCL. Selection of the optimal treatment regimen is challenging and warrants further research.
